# Food Insecurity Among Patients with Chronic Pain in an Urban Slum of Delhi: A Community-Based Cross-Sectional Study

**DOI:** 10.3390/nu18193185

**Published:** 2026-09-26

**Authors:** Shivani Rao, Kinshuk Gupta, Priyanka Aggarwal, Amod Laxmikant Borle, Mongjam Meghachandra Singh, Nandini Sharma

**Affiliations:** 1 Department of Community Medicine, Maulana Azad Medical College, New Delhi 110002, India; shivani.idsp@gmail.com (S.R.); priyanka711.pa@gmail.com (P.A.); amodborle@gmail.com (A.L.B.); megharita1@gmail.com (M.M.S.); 2 Independent Researcher, New Delhi 110002, India; drnandini1@gmail.com

**Keywords:** chronic pain, food insecurity, food insecurity experience scale, dietary diversity, urban slum, India

## Abstract

**Background:** Food insecurity, defined as a lack of consistent access to sufficient, safe, and nutritious food, is an important social determinant of health. However, its relationship with chronic pain remains poorly understood, particularly in low-and middle-income (LMIC) countries. This study aims to estimate the prevalence of food insecurity and identify its associated factors among patients with chronic pain in an urban slum of Delhi, India. **Methods:** A community-based cross-sectional study was conducted in the urban field practice area of Gokalpuri, North-East Delhi. A total of 151 adults aged ≥18 years with chronic pain persisting for more than three months were selected through simple random sampling from a household register. Food insecurity was assessed using the validated Food Insecurity Experience Scale (FIES). Sociodemographic, clinical, economic, psychological and nutritional characteristics were assessed using a semi-structured questionnaire. A priori selected variables were entered simultaneously into a multivariable logistic regression model. **Results:** The mean age of participants was 53.0 years, and 74.2% were women. Overall, 41.1% of participants were food insecure, including 16.6% with mild, 8.0% with moderate, and 16.6% with severe food insecurity. Dietary diversity was inadequate among 86.8% of participants which worsened with increasing food insecurity severity (*p* = 0.005). In the multivariable logistic regression analysis, cost-related care-seeking delay (OR = 10.10, 95% CI: 2.80–36.47, *p* < 0.001) was independently associated with higher odds of food insecurity, while adequately controlled pain (OR = 0.35, 95% CI: 0.16–0.77, *p* = 0.009) was independently associated with lower odds. Food-insecure participants also reported significantly poorer wellbeing than food-secure participants (37.1% vs. 60.7% adequate wellbeing, respectively; χ^2^ = 8.13, *p* = 0.004). **Conclusions:** Food insecurity affected two in five individuals with chronic pain. Cost-related delays in care-seeking were associated with increased odds of food insecurity, whereas adequate pain control was associated with reduced odds. Food-insecure participants also reported poorer wellbeing. Nutritional vulnerability in this population warrants greater attention as part of a comprehensive approach to chronic pain management.

## 1. Introduction

Chronic pain has emerged as a significant public health challenge in LMICs such as India, with prevalence ranging from 19.2% to 62% across populations, settings and various case definitions in India [1,2,3,4]. Beyond its immediate physical impact, it causes psychosocial distress, stigma, social isolation, and economic hardship, affecting individuals, families, communities, and health systems [1,5,6]. It arises from a complex interplay of biological, psychological, behavioural, sociocultural, and environmental factors [7], among which nutrition has gained increasing attention as a potentially modifiable contributor. It exerts its influence on inflammatory, immune, and metabolic pathways that are implicated in pain development and persistence [8,9].

Food insecurity is defined as the lack of consistent access to sufficient, safe, and nutritious food for an active and healthy life [10]. Indians continue to suffer from it despite economic progress, with approximately 50% of socially disadvantaged Indians still experiencing food insecurity [11]. Food quality, on the other hand, reflects the nutritional composition of what is actually consumed. A growing body of evidence has associated food insecurity with an increased risk of pain and pain-related emergency room visits [12,13]. In another study from the United States, more than half of food-insecure individuals reported chronic pain [14]. Recent research suggests that food insecurity may be a stronger predictor of chronic pain than conventional socioeconomic indicators [13,15]. Poor nutrient intake and insufficient food consumption have been linked to the development and persistence of chronic noncancer pain, plausibly through sustained low-grade inflammation and heightened nociceptive sensitivity [9,16,17]. Food insecurity has also been associated with immune dysregulation and mental health difficulties, including anxiety and depression, which may further increase vulnerability to pain and disability [13,14].

People suffering from chronic pain develop food access problems through multiple connected pathways. The functional impairment can restrict an individual’s capacity to work, shop for, and prepare food. The substantial cost of treatment can compete with household spending on food. It may interact bidirectionally with nutritional status through inflammatory and psychosocial pathways [7,16,17].

However, the relationship between chronic pain and food insecurity remains understudied. Existing epidemiological evidence is largely derived from high-income settings such as the United States and Canada, which have different safety nets, healthcare financing, and dietary patterns than those found in low- and middle-income countries.

Unlike previous Indian studies that have examined food insecurity in the general population or among older adults broadly, this study specifically targets a chronic pain population, thereby generating context-specific evidence to inform integrated pain and nutrition-sensitive care in resource-limited urban settings. Further, urban slum populations experience a heightened risk of food insecurity due to poverty, precarious informal employment, inadequate social protection, and substandard living conditions [18,19]. Therefore, we conducted a community-based cross-sectional study to estimate the prevalence of food insecurity and identify its associated factors among patients with chronic pain in an urban slum of Delhi.

## 2. Materials and Methods

### 2.1. Study Design and Setting

This was a community-based cross-sectional study conducted in the urban field practice area of Gokalpuri, North-East Delhi, India. Gokalpuri is a densely populated, lower-middle-income urban locality representative of peri-urban Delhi. The area is served by the Urban Health Training Centre of the Department of Community Medicine and maintains a household register that formed the sampling frame for this study. The study area is characterised by mixed residential and commercial zones, with a predominantly lower-middle-income population engaged in informal and small-scale employment.

### 2.2. Study Population

Participants were eligible if they were (i) permanent residents of the Gokalpuri field practice area for at least six months, (ii) aged 18 years or above. Chronic pain was defined using the Graded Chronic Pain Scale–Revised (GCPS-R) [20] as pain persisting for more than three months and occurring on most or all days. Participants requiring healthcare were advised to seek appropriate care at the Urban Health Training Centre. Individuals who were unable to understand the questionnaire adequately were excluded from the study.

### 2.3. Sample Size and Sampling

Sample size was calculated using the formula $n=Z^{2}p(1-p)/d^{2}$, assuming a prevalence of food insecurity of 45.84% from previous Indian literature [11], a 95% confidence level (Z = 1.96), and 8% absolute precision (d). The minimum required sample size was estimated to be 149, which was rounded to 151 participants. Participants were recruited using simple random sampling. A sampling frame was constructed from the household register of the urban field practice area maintained by the Department of Community Medicine. Households with at least one eligible participant were identified through a preliminary household survey from November 2025 to January 2026, in which field investigators visited households listed in the register and screened all resident adults according to the inclusion criteria. A random number sequence was generated using a computer-based random number generator, and households were selected in the order of their assigned random numbers. Where more than one eligible participant was present in a household, one participant was selected using the Kish grid method to avoid clustering of multiple participants from the same household. In total, 1500 households were screened, of which 158 (10.5%) contained at least one eligible individual with chronic pain; 1342 households (89.5%) had no eligible participant. Of the 158 eligible individuals, 7 (4.4%) declined participation, yielding a final analytic sample of 151 (95.6% of those eligible). The detailed interviews for the eligible participants were conducted from February 2026 to April 2026. The participant recruitment flow is shown in Figure 1.

The semi-structured questionnaire was developed by the investigators following a review of the published literature and relevant validated instruments. It was pilot-tested among 15 individuals with chronic pain from the study area who were not included in the final analysis. The pilot study assessed the clarity, comprehensibility, cultural appropriateness, and acceptability of the questionnaire, including the Hindi translation, as well as the feasibility of administration. Based on participant feedback and field observations, the questionnaire was refined before the commencement of the main study.

All field investigators underwent a structured training programme conducted by the principal investigator prior to commencement of data collection. Training covered: (i) detailed familiarisation with the study questionnaire and interview procedures, including probing techniques; (ii) standardised anthropometric measurement procedures with inter-rater reliability testing; (iii) informed consent procedures; and (iv) data recording and quality assurance.

To ensure consistency and data quality throughout the study, the principal investigator provided ongoing field supervision, and a random subset of completed interviews was re-contacted by telephone or repeat household visit to verify key responses. Electronically collected data were reviewed daily for completeness, internal consistency, and range errors, with discrepancies clarified with the field investigator concerned before the next round of data collection.

### 2.4. Outcome Variable

Food insecurity was assessed using the Food Insecurity Experience Scale (FIES) [21], a validated eight-item tool. The Food Insecurity Experience Scale (FIES) was administered using the Hindi version, which is available as an official language-adapted version of the FAO FIES and has been used in Indian settings.

The eight-item FIES demonstrated good internal consistency (Cronbach’s α = 0.88) and a unidimensional structure. The first factor had an eigenvalue of 4.60 and explained 57.5% of the total variance, with factor loadings ranging from 0.61 to 0.81. The overall KMO measure of sampling adequacy was 0.849.

Participants were categorised based on the standard FAO Voices of the Hungry classification scheme [21]: food secure, mildly food insecure, moderately food insecure, and severely food insecure. Mild food insecurity referred to concerns regarding food availability or compromise in dietary quality; moderate food insecurity referred to reduction in food intake quantity, skipping meals, or inability to consume balanced meals; while severe food insecurity referred to experiences of hunger, going without food, or not eating for an entire day due to lack of resources.

### 2.5. Predictor Variable

Candidate predictor variables were selected a priori across five conceptual domains based on published literature and their relevance to chronic pain in a resource-constrained urban setting. Detailed household food expenditure, household debt or borrowing history, and detailed dietary intake were considered but not collected due to recall concerns, assessment complexity, and respondent burden.

Sociodemographic domain: Age, gender, occupation, marital status, family type, educational status, and socioeconomic status were assessed using a semi-structured questionnaire. Socioeconomic status was classified using the updated Modified B.G. Prasad socioeconomic classification scale [22].

Clinical domain: Pain intensity was assessed using the 11-point Numeric Rating Scale [23] and pain location was assessed using the Standardized Nordic Musculoskeletal Questionnaire body map [24]. Perceived causes of chronic pain, pain frequency, and the number of tablets consumed for pain relief were assessed using a semi-structured questionnaire. Pain control was assessed by participants’ self-report of whether their current treatment provided adequate pain relief. Multisite pain was defined as pain involving more than one anatomical region. Polypharmacy was defined as the concurrent use of five or more medications [25].

Economic domain: Perceived treatment affordability and cost-related care-seeking delay during the preceding month were assessed using a semi-structured questionnaire.

Nutritional domain: Body mass index was calculated and classified according to World Health Organization criteria [26]. Anaemia status was determined by self-reported physician diagnosis during the preceding three months, based on World Health Organization haemoglobin cut-offs [27]. Dietary diversity was assessed using a questionnaire adapted from the Individual Dietary Diversity Score framework and the Minimum Dietary Diversity for Women (MDD-W) tool [28,29]. We adapted the underlying food-group framework to assess individual dietary diversity by incorporating locally relevant food examples and dietary practices in the study setting. This approach has previously been used due to unavailability of validated indicators for specific sub-groups [30]. Those who consumed five or more food items in the previous day were considered to have adequate diversity.

Psychological domain: Well-being, depression, anxiety, sleep quality, and disability were assessed using the WHO-5 Well-Being Index [31], Patient Health Questionnaire-2 [32], Generalized Anxiety Disorder-2 [33], Single-Item Sleep Quality Scale [34], and the 12-item World Health Organization Disability Assessment Schedule 2.0 [35], respectively. WHO-5 scores were converted to a 0–100 scale, with scores > 50% classified as adequate well-being and ≤50% as poor well-being. A score of ≥3 on the PHQ-2 and GAD-2 was considered indicative of depression and anxiety, respectively. Sleep quality was classified as poor for scores ≥ 6. WHODAS 2.0 scores were converted to a 0–100 scale, with scores ≥ 25% classified as significant disability.

### 2.6. Statistical Analysis

All data were collected electronically using KoboCollect Toolbox (KoBoToolbox, Harvard Humanitarian Initiative, Cambridge, MA, USA), exported into a structured spreadsheet, cleaned for inconsistencies and range errors, and analysed using Stata version 19 BE (StataCorp LLC, College Station, TX, USA). Prior to analysis, the dataset was examined for missing values.

Continuous variables were assessed for normality using the Shapiro–Wilk test. Normally distributed variables are reported as mean ± standard deviation (SD), while non-normally distributed variables are presented as median (interquartile range, IQR). Categorical variables are reported as frequency and percentage. Linear trends across ordered categories of food insecurity were evaluated using the Cochran-Armitage test for trend. Associations between categorical predictors and food insecurity status were assessed using the Chi-square test or Fisher’s exact test as appropriate.

For the multivariable regression analysis, food insecurity was dichotomised as food secure versus food insecure (combining mild, moderate, and severe categories). This approach was adopted primarily because of the relatively small number of participants in each category, which would have limited the stability and precision of category-specific estimates in an ordinal model. Six predictor variables—pain control, socioeconomic status, age group, gender, cost-related care-seeking delay, and occupation—were selected a priori, on the basis of their established or plausible clinical and policy relevance to food insecurity in a chronic-pain population, and were entered simultaneously into a single multivariable logistic regression model using a forced-entry approach. Adjusted odds ratios (aORs) with 95% confidence intervals (CIs) were reported. Multicollinearity among predictor variables was assessed using the variance inflation factor (VIF), with a VIF > 5 considered indicative of problematic collinearity. Overall model significance was evaluated using the likelihood-ratio χ^2^ test, model fit was summarised using McFadden’s pseudo R^2^, Akaike’s Information Criterion (AIC), and the Bayesian Information Criterion (BIC), and discriminatory performance was assessed using the area under the receiver operating characteristic curve (AUC). A two-tailed *p*-value < 0.05 was considered statistically significant.

### 2.7. Ethical Approval

The study was conducted in accordance with the ethical principles laid down in the Declaration of Helsinki (2013 revision). Ethical approval was obtained from the Institutional Ethics Committee of the parent institution prior to commencement of fieldwork (IEC/MAMC/116/04/2025/439). Written informed consent was obtained from all participants after explaining the study objectives, procedures, voluntary nature of participation, confidentiality safeguards, and the right to withdraw at any stage without consequence. All data were anonymised prior to analysis, and no personally identifiable information was included in the final dataset.

## 3. Results

### 3.1. Sociodemographic Characteristics of Participants

Complete data were available for all 151 participants for all sociodemographic, functional, economic, and nutritional variables assessed. Table 1 depicts the sociodemographic profile of the study participants. The mean age of the participants was 53.03 years, with ages ranging from 22 to 76 years. The highest proportion of participants belonged to the 50–59-year age group (32.5%), followed by those aged 40–49 years and 60–69 years. Females comprised nearly three-fourths of the participants (74.2%). Most participants were married (84.1%), belonged to nuclear families (72.8%) and were Hindu by religion (97.4%). Primary education was the most commonly reported category (34.4%), while 27.2% of participants were illiterate. Homemakers constituted the largest occupational group (68.9%). Nearly half of the participants belonged to the upper middle class (45.7%).

### 3.2. Pain Characteristics

Slightly over half of participants (52.3%) reported adequate pain control(Table 2). Mean pain intensity was 5.3 ± 2.2, with 19.2% reporting daily pain. Multisite pain was reported by 98 (64.9%) participants, with a median of 2 painful sites (IQR: 1–3). The feet and ankles were the most commonly affected region (48.3%). Participants used a median of 3 pain medications (IQR: 2–4), with nutritional factors (46.4%) the most common perceived contributor. 

### 3.3. Food Insecurity and Dietary Diversity Among Study Participants

More than two out of five participants were food insecure (41.1%). Mild and severe food insecurity were each reported by 16.6% of participants, while moderate food insecurity was observed among 8.0% of the study population.

Food insecurity was more common among females than males across all severity categories (Figure 2). A significant linear trend was observed across food insecurity categories (*p* = 0.009).

Participants with inadequate dietary diversity had a substantially higher prevalence of food insecurity across all severity levels compared with those with adequate dietary diversity. (Figure 3) A significant linear trend was observed across food insecurity severity categories (*p* = 0.005).

### 3.4. Bivariate Analysis

Food insecurity was more prevalent among females than males (46.4% vs. 25.6%; *p* = 0.023) and among homemakers than working or retired participants (*p* = 0.024). No significant associations were observed with age, marital status, family type, education, or socioeconomic status. In the clinical domain, food insecurity was more common among those with inadequately controlled pain (52.8% vs. 30.4%; *p* = 0.002), while multisite pain was not associated (*p* = 0.792). No significant associations were observed with depression, anxiety, sleep quality, disability, or anaemia. Food insecurity was strongly associated with cost-related care-seeking delay (*p* < 0.001), perceived treatment unaffordability (*p* = 0.001), inadequate dietary diversity (*p* = 0.003), and BMI category (*p* = 0.035) (Table 3).

Six predictor variables were entered simultaneously into a multivariable logistic regression model (Table 4). No evidence of problematic multicollinearity was observed among the included predictors, with all variance inflation factors below 5. The overall model was statistically significant (LR χ^2^(6) = 38.67, *p* < 0.001), with an AUC of 0.778, indicating acceptable discriminatory ability. Participants reporting cost-related care-seeking delay had approximately ten-fold higher odds of food insecurity than those without treatment delay (OR = 10.10, 95% CI: 2.80–36.47, *p* < 0.001), and those with adequately controlled pain had 65% lower odds of food insecurity than those with inadequately controlled pain (OR = 0.35, 95% CI: 0.16–0.77, *p* = 0.009), consistent with the effect reported in the abstract and conclusions.

### 3.5. Food Security and Wellbeing

Participants who were food secure were significantly more likely to report adequate wellbeing than those who were food insecure (60.7% vs. 37.1%, respectively), while poor wellbeing was correspondingly more common among food-insecure participants (62.9% vs. 39.3%; χ^2^ = 8.13, *p* = 0.004) (Table 5).

## 4. Discussion

This community-based cross-sectional study is among the first from India to characterise food insecurity specifically within a chronic pain population, and demonstrates that it is a substantial and independent concern in this group. Conducted in an urban slum, it found the prevalence of food insecurity among patients with chronic pain to be 41.1%, with 16.6% mild, 8.0% moderate, and 16.6% severe food insecurity. Inadequate dietary diversity was highly prevalent (86.8%) and showed a significant trend with food insecurity severity. Food-insecure participants also reported significantly poorer wellbeing than food-secure participants. In the multivariable model, cost-related care-seeking delay and inadequately controlled pain were independently associated with food insecurity.

Previous Indian studies have reported widely varying prevalence estimates of food insecurity, ranging from 8.5% to 48.8%, owing to differences in operational definitions and study population [36,37,38]. The prevalence observed in our study is comparable to that reported among residents of Delhi slums by Joshi et al., but higher than estimates from other slum-based studies (30.1%) [18,19]. The comparatively higher prevalence observed in our study may be explained by the fact that our study population consisted entirely of individuals suffering from chronic pain, a condition associated with persistent healthcare expenditure, disability, reduced productivity, and long-term financial strain. It remains lower than pooled estimates from Southern Asia, where 36.5% of people were reported to be moderately food insecure, as well as lower-middle-income countries such as Iran (59.1%) and the Philippines (65%) [10,39].

Cost-related care-seeking delay was the strongest independent correlate of food insecurity in our multivariable model, associated with approximately ten-fold higher odds (OR = 10.10, 95% CI: 2.80–36.47, *p* < 0.001). This finding is consistent with previous studies identifying economic vulnerability as a key correlate of food insecurity [40,41,42]. Chronic pain is associated with substantial direct and indirect costs, including healthcare expenditure, loss of productivity, and reduced earning capacity, which can lead to catastrophic health expenditure and impoverishment of vulnerable households [42,43,44].

Adequately controlled pain was independently associated with 65% lower odds of food insecurity relative to inadequately controlled pain. Although research directly examining this association is limited, our findings are broadly consistent with existing evidence linking a higher burden of chronic pain to worsening food insecurity. However, both these pathways need to be cautiously interpreted due to the cross-sectional design. Moreover, both controlled pain status and cost-related care-seeking may reflect overlapping barriers to healthcare access. Thus, reverse causation and residual confounding by unmeasured economic or health-system factors cannot be excluded.

The association between dietary diversity and food insecurity severity was strong and showed a significant linear trend across categories. While no previous study has specifically examined this relationship among individuals with chronic pain, the broader literature consistently describes a bidirectional relationship between food insecurity and dietary diversity [45]. Individuals experiencing food insecurity often prioritise inexpensive, energy-dense foods due to their affordability and easy availability [46], which can result in monotonous diets lacking in essential micronutrients and antioxidants. Such diets have been linked to chronic low-grade inflammation, poorer nutritional status, and greater pain severity. We propose this as one plausible self-perpetuating cycle linking food insecurity, poor dietary diversity, and chronic pain; however, the inflammatory and nutritional pathways themselves were not directly measured in this study.

The high prevalence of food insecurity and inadequate dietary diversity observed in this study highlights an important but often overlooked dimension of chronic pain. Nutritional vulnerability in this population warrants greater attention as part of a comprehensive approach to chronic pain management, particularly in resource-constrained settings.

Multiple policy implications emerge from these findings. Routine screening for food insecurity and cost-related care-seeking delay could usefully be integrated into chronic pain management in primary care and community health settings. Linking food-insecure patients with chronic pain to existing social protection and food-support schemes may help interrupt the cycle of pain, economic hardship, and poor nutrition described above. However, these need further testing in form of prospective and longitudinal designs to clarify the temporal and potentially bidirectional relationship between chronic pain, food insecurity, and wellbeing.

The present study has several strengths. To the best of our knowledge, it is among the first community-based studies from India to examine food insecurity among individuals with chronic pain. Multidimensional assessment, use of validated tools, and community-based sampling strengthen the validity and relevance of the findings.

Several limitations should be acknowledged. The cross-sectional design precludes establishing temporal or causal relationships, and reverse causation cannot be excluded. The study was conducted in a single urban slum with a relatively small sample, limiting the generalisability of the findings beyond the study population. Although random sampling was used, the analytic sample was predominantly female, which may affect the representativeness of the sample and the generalisability of sex-specific findings. Contemporaneous population-level data for Gokulpuri were unavailable, limiting our ability to assess the representativeness and generalisability of the findings. Several adjusted estimates had wide confidence intervals, indicating uncertainty in the magnitude of the observed associations. Finally, although most study measures used validated instruments, the dietary diversity assessment was adapted from the MDD-W, which was developed and validated for women of reproductive age. Its application to our mixed-sex, mixed-age population may limit its applicability, and the findings should therefore be interpreted as reflecting dietary variety rather than validated dietary adequacy.

## 5. Conclusions

This community-based cross-sectional study of 151 patients with chronic pain in an urban slum of Delhi found that 41.1% of participants were food insecure. Inadequate dietary diversity was highly prevalent and showed a significant trend with food insecurity severity. Food-insecure participants also reported significantly poorer wellbeing than food-secure participants. In a multivariable model, cost-related care-seeking delay was independently associated with higher odds of food insecurity, while adequately controlled pain was independently associated with lower odds. These findings should be interpreted with appropriate caution given the cross-sectional design and the possibility of reverse causation and residual confounding. The high prevalence of food insecurity and inadequate dietary diversity observed in this study suggests that nutritional vulnerability may be common among individuals with chronic pain in resource-constrained settings. Prospective longitudinal and intervention studies are needed to clarify the direction of these associations, including the relationship between food security and wellbeing, and to determine whether improving food security can influence pain-related outcomes, and vice versa.

## Figures and Tables

**Figure 1:**
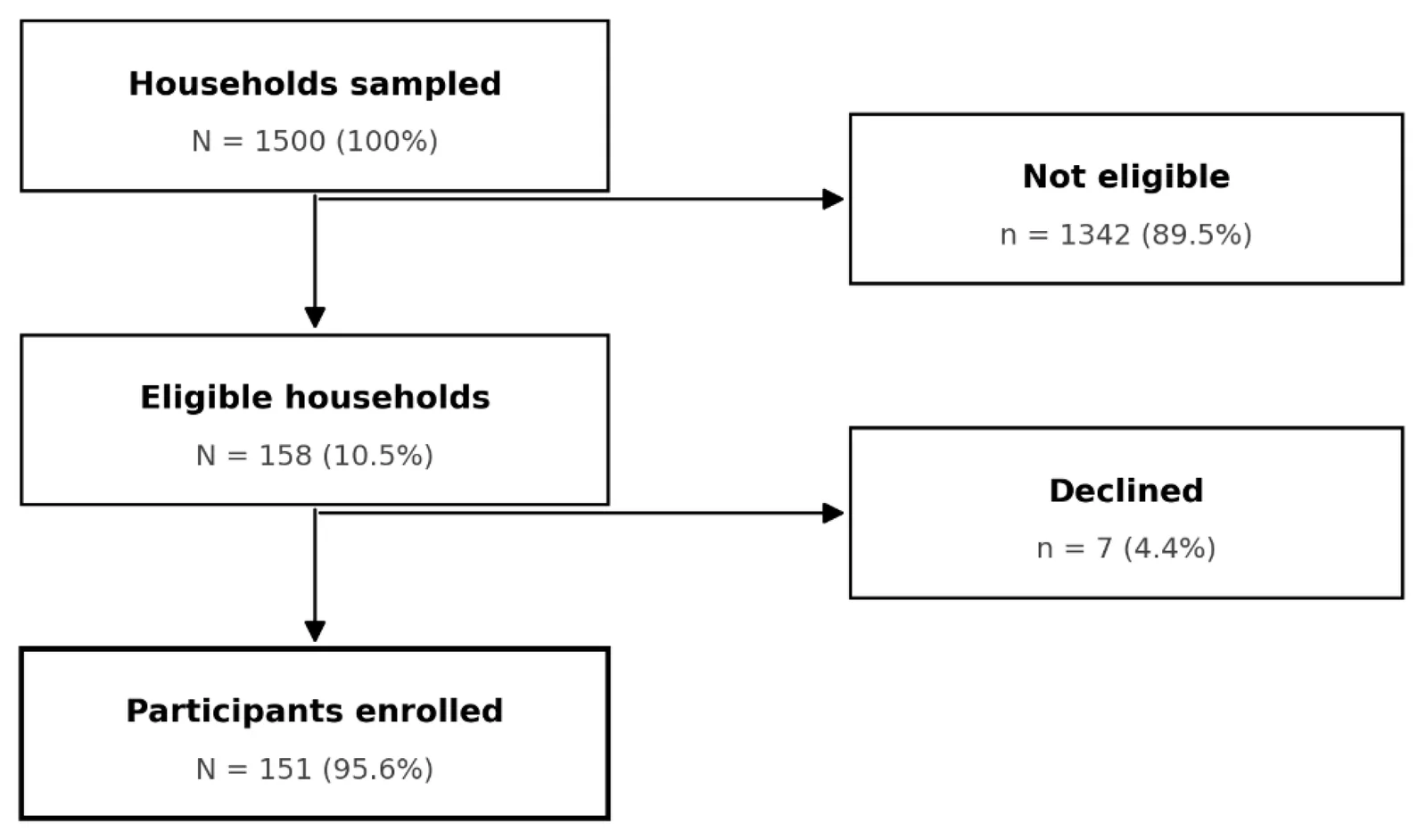
Participant recruitment flow.

**Figure 2:**
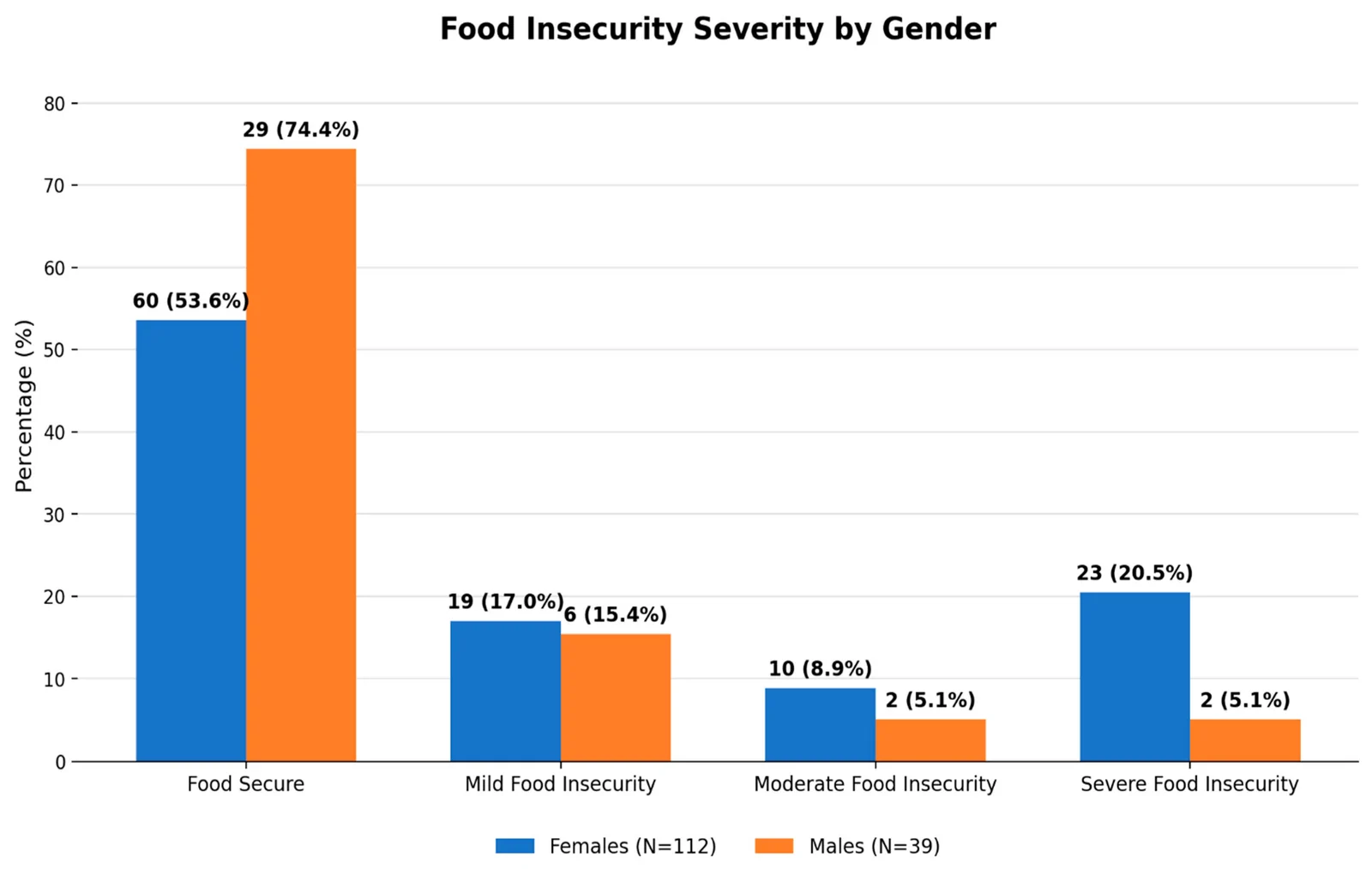
Gender-disaggregated distribution of participants according to food insecurity status.

**Figure 3:**
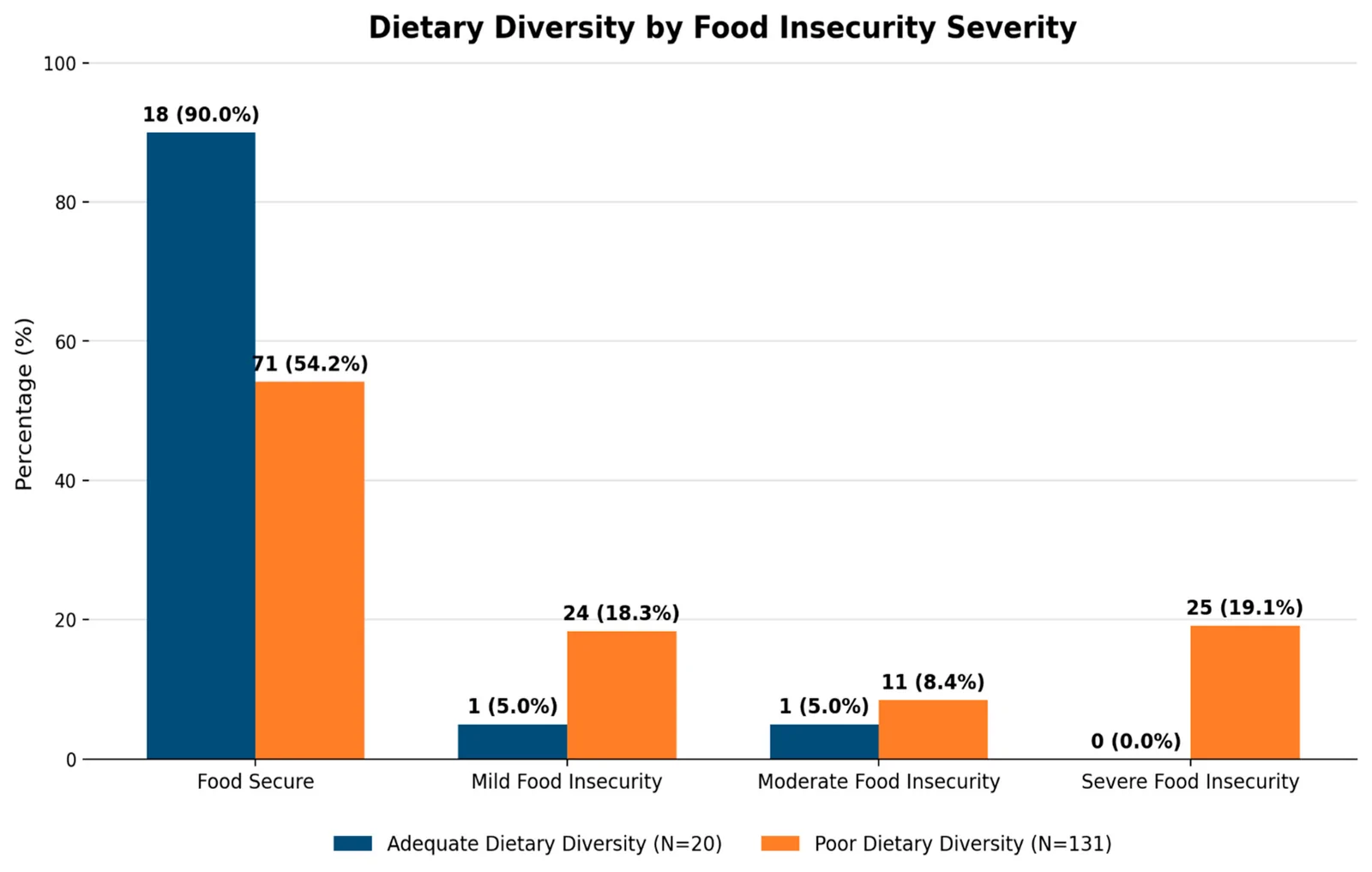
Distribution of dietary diversity according to food insecurity status.

**Table 1:** Sociodemographic characteristics of study participants (N = 151).

Variable	n (%)
Age	
<40 years	15 (9.9)
40–49 years	37 (24.5)
50–59 years	49 (32.5)
60–69 years	37 (24.5)
≥70 years	13 (8.6)
Gender	
Female	112 (74.2)
Male	39 (25.8)
Occupation	
Homemaker	104 (68.9)
Retired	31 (20.5)
Working	16 (10.6)
Marital Status	
Married	127 (84.1)
Divorced/Widowed/Separated	21 (13.9)
Single	3 (2.0)
Family Type	
Nuclear family	110 (72.8)
Extended family	24 (15.9)
Joint family	17 (11.3)
Educational Status	
Illiterate	41 (27.2)
Primary education	52 (34.4)
Middle school	15 (9.9)
High school	25 (16.6)
Diploma/Intermediate	13 (8.6)
Graduate	4 (2.7)
Postgraduate	1 (0.7)
Socioeconomic Status	
Lower class	8 (5.3)
Lower middle class	24 (15.9)
Middle class	45 (29.8)
Upper middle class	69 (45.7)
Upper class	5 (3.3)
Religion	
Hindu	147 (97.4)
Muslim	1 (0.7)
Other religion	3 (2.0)

**Table 2:** Clinical characteristics of chronic pain among study participants (N = 151).

Variable	n (%)
Pain site *	
Feet and ankles	73 (48.3)
Knees	72 (47.7)
Lower back	64 (42.4)
Upper back	40 (26.5)
Shoulders	31 (20.5)
Neck	21 (13.9)
Elbows	18 (11.9)
Hips/thigh	16 (10.6)
Wrists and hands	8 (5.3)
Perceived reasons for pain *	
Nutritional factors	70 (46.4)
Sleep-related factors	61 (40.4)
Occupational/physical activities	48 (31.8)
Age-related changes	37 (24.5)
Treatment-related factors	32 (21.2)
Domestic workload	24 (15.9)
Weather-related factors	24 (15.9)
No perceived cause	14 (9.3)
Physical inactivity	7 (4.6)
Previous trauma	4 (2.6)

* Multiple responses permitte.

**Table 3:** Factors associated with food insecurity among patients with chronic pain (N = 151).

Variable	Category	N	Food Insecure n (%)	Food Secure n (%)	Test	*p*-Value
**Sociodemographic domain**						
Gender	Female	112	52 (46.4)	60 (53.6)	Chi^2^	0.023 *
	Male	39	10 (25.6)	29 (74.4)		
Age group	<50 years	52	17 (32.7)	35 (67.3)	Chi^2^	0.130
	≥50 years	99	45 (45.5)	54 (54.5)		
Occupation	Homemaker	104	49 (47.1)	55 (52.9)	Chi^2^	0.024 *
	Retired/Working	47	13 (27.7)	34 (72.3)		
Marital status	Married	127	51 (40.2)	76 (59.8)	Chi^2^	0.604
	Single/Divorced/Widowed	24	11 (45.8)	13 (54.2)		
Family type	Nuclear	110	46 (41.8)	64 (58.2)	Chi^2^	0.756
	Joint/Extended	41	16 (39.0)	25 (61.0)		
Educational status	Any formal education	110	43 (39.1)	67 (60.9)	Chi^2^	0.421
	Illiterate	41	19 (46.3)	22 (53.7)		
Socioeconomic status	Middle/Upper-middle/Upper	119	46 (38.7)	73 (61.3)	Chi^2^	0.247
	Lower & Lower-middle	32	16 (50.0)	16 (50.0)		
Clinical domain						
Pain control	Adequately controlled	79	24 (30.4)	55 (69.6)	Chi^2^	0.002 *
	Inadequately controlled	72	38 (52.8)	34 (47.2)		
Multisite pain	Absent	53	21 (39.6)	32 (60.4)	Chi^2^	0.792
	Present	98	41 (41.8)	57 (58.2)		
Polypharmacy	Absent	116	52 (44.8)	64 (55.2)	Chi^2^	0.113
	Present	35	10 (28.6)	25 (71.4)		
Psychological domain						
Depression	Negative screen	63	27 (42.9)	36 (57.1)	Chi^2^	0.704
	Positive screen	88	35 (39.8)	53 (60.2)		
Anxiety	Negative screen	63	25 (39.7)	38 (60.3)	Chi^2^	0.771
	Positive screen	88	37 (42.0)	51 (58.0)		
Sleep quality	Good	53	18 (34.0)	35 (66.0)	Chi^2^	0.192
	Poor	98	44 (44.9)	54 (55.1)		
Disability	No significant disability	82	31 (37.8)	51 (62.2)	Chi^2^	0.375
	Significant disability	69	31 (44.9)	38 (55.1)		
Economic domain						
Perceived treatment affordability	Affordable	116	39 (33.6)	77 (66.4)	Chi^2^	0.001 *
	Not affordable	35	23 (65.7)	12 (34.3)		
Cost-related care-seeking delay	Absent	35	3 (8.6)	32 (91.4)	Chi^2^	<0.001 *
	Present	116	59 (50.9)	57 (49.1)		
Nutritional domain						
Anaemia	Absent	139	57 (41.0)	82 (59.0)	Chi^2^	0.964
	Present	12	5 (41.7)	7 (58.3)		
BMI category	Underweight/Normal	58	30 (51.7)	28 (48.3)	Chi^2^	0.035 *
	Overweight/Obese	93	32 (34.4)	61 (65.6)		
Dietary diversity	Inadequate	131	60 (45.8)	71 (54.2)	Fisher’s exact	0.003 *
	Adequate	20	2 (10.0)	18 (90.0)		

N = 151. Chi^2^ = Pearson chi-square test. * *p* < 0.05.

**Table 4:** Multivariable logistic regression—independent factors of food insecurity among patients with chronic pain (N = 151).

Variable	Category	Unadj. OR	95% CI	*p*	Adj. OR	95% CI	*p*
Pain control	Controlled vs. Uncontrolled	0.35	0.18–0.68	0.002 *	0.35	0.16–0.77	0.009 *
Socioeconomic status	Middle/Lower vs. Upper	1.81	0.94–3.50	0.076	1.22	0.56–2.69	0.618
Age group	≥50 years vs. <50 years	1.72	0.85–3.46	0.132	1.56	0.68–3.56	0.293
Gender	Male vs. Female	0.40	0.18–0.89	0.026 *	0.27	0.05–1.47	0.129
Cost-related care-seeking delay	Present vs. Absent	11.04	3.20–38.08	<0.001 *	10.10	2.80–36.47	<0.001 *
Employment status	Retired/Working vs. Homemaker	0.43	0.20–0.91	0.026 *	1.25	0.25–6.14	0.786

Model fit: LR χ^2^(6) = 38.67, *p* < 0.001|McFadden’s pseudo R^2^ = 0.189|AIC = 179.81|BIC = 200.93|AUC = 0.778|N = 151. Notes: Unadjusted ORs were obtained from univariable logistic regression models. Adjusted ORs were estimated from the multivariable logistic regression model. Reference categories: Pain control = Uncontrolled pain; Socioeconomic status = Upper; Age group = <50 years; Gender = Female; Cost-related care-seeking delay = Absent; Employment status = Homemaker. OR = Odds Ratio; CI = Confidence Interval. * *p* < 0.05.

**Table 5:** Association between food security status and wellbeing (secondary, exploratory analysis; N = 151).

Food Security Status	N	Adequate Wellbeing n (%)	Poor Wellbeing n (%)	Test	*p* Value
Food secure	89	54 (60.7)	35 (39.3)	Chi^2^	0.004 *
Food insecure	62	23 (37.1)	39 (62.9)		

* *p* < 0.05.

## Data Availability

The data presented in this study are available from the corresponding author upon reasonable request. The data are not publicly available due to ethical and privacy restrictions.
